# Monitoring and Statistical Analysis of Formation of Organochlorine and Organobromine Compounds in Drinking Water of Different Water Intakes

**DOI:** 10.3390/molecules26071852

**Published:** 2021-03-25

**Authors:** Margarita Yu. Vozhdaeva, Alfiya R. Kholova, Igor A. Melnitskiy, Ilya I. Beloliptsev, Yulia S. Vozhdaeva, Evgeniy A. Kantor, Albert T. Lebedev

**Affiliations:** 1 State Unitary Enterprise “Ufavodokanal”, Water Treatment Station, Rossiyskaya St. 157/2, Ufa 450098, Russia; al-pochta@mail.ru (A.R.K.); i_melnit@rambler.ru (I.A.M.); 2 Department of Petrochemistry and Chemical Technology, Department of Physics, Ufa State Petroleum Technical University, Kosmonavtov St. 1, Ufa 450000, Russia; evgkantor@mail.ru; 3 Department of Mathematics and Computer Science, Ufa Branch, Financial University, Mustaia Karima St. 69/1, Ufa 450015, Russia; IIBeloliptsev@fa.ru; 4 Department of Mechanics and Mathematics, St. Petersburg State University, Universitetskaia Emb. 7/9, Saint-Petersburg 199034, Russia; uni_vozhd@mail.ru; 5 Organic Chemistry Department, Lomonosov Moscow State University, Leninskie Gori 1/3, Moscow 119991, Russia

**Keywords:** brominated DBP, chlorinated DBP, THM, HAA, SVOC, GC-MS, aqueous chlorination

## Abstract

The main drawback of drinking water chlorination involves the formation of quite hazardous disinfection by-products (DBPs), represented mainly by halogenated species. Based on the authors’ monitoring data since 2002, the prevalence of chlorine over bromine in the composition of volatile DBPs was shown for the drinking water in Ufa (Russia). However, the situation was completely reversed in the case of semi-volatile DBPs. The principal goal of the present study involved rationalization of the results of the long-term monitoring. Gas chromatography–mass spectrometry (GC-MS) was used for the qualitative and quantitative analysis of volatile DBPs. Identification of semi-volatile compounds was carried out with GC-MS, while gas chromatography with an atomic emission detector (GC-AED) was used for their quantification. A significant contribution of oxygen to the composition of semi-volatile compounds proves the decisive role of the dissolved organic matter oxidative destructive processes. Statistical analysis revealed notable linear correlations for trihalomethane and haloacetic acid formation vs. chlorine dose. On the contrary, halogenated semi-volatile products do not demonstrate any correlations with the water quality parameters or chlorine dose. Principal component analysis (PCA) placed them into separate groups. The results allow for proposing that formation of the organohalogenated species involved the fast penetration of bromine into the humic matter molecules and, further, their oxidative destruction by active chlorine.

## 1. Introduction

The quality of drinking water dramatically influences human health. Drinking water should satisfy the required hygienic standards, and disinfection represents one of the most important stages of water preparation and the most efficient tool to prevent diseases spreading through water [1]. Elimination of pathogenic microorganisms during water preparation started at the beginning of the 20th century and considerably decreased the threat of these diseases [2]. Chlorination remains the most widespread method of water disinfection all over the world. A critical advantage of chlorination over alternative methods of disinfection involves its prolonged activity, which is crucial for big cities with extended water distribution networks, while its main drawback is the formation of numerous disinfection by-products (DBPs). The study of the formation of trihalomethanes (THMs) and haloacetic acids (HAAs) as the major DBPs started in the 1970s [3,4,5,6,7,8,9]. Nevertheless, organohalogenated compounds of a higher mass (aldehydes, ketones, acids, acetonitriles, phenols, etc.) may also be quite hazardous [10,11,12,13,14]. Over 700 DBPs are currently known [15], constituting only half of the total of organic chlorine. Natural dissolved organic matter (humic and fulvic acids, phenols, amines, products of algal metabolism) is the principal precursor of DBPs [16,17,18,19,20,21,22]. Intensive anthropogenic impacts on drinking water sources lead to the inhibition of natural water self-purification processes. It may be accompanied by an increase in the abundance and assortment of phytoplankton and aquatic plants, resulting in humification [23]. Wastage of a notable portion of the dissolved oxygen on the oxidation of that additional organic matter induces an oxygen deficit and intensification of eutrophication. These changes lead to corresponding changes in the qualitative and quantitative composition of the chlorination by-products in drinking water. Anthropogenic impacts have an additional consequence. More and more industrial products appear in natural water and react with disinfecting agents at water treatment stations, producing novel DBPs [24,25,26,27,28,29].

Peculiarities of the aquatic chlorination depending on the characteristics of humic matter have attracted significant attention from the scientific community [30,31,32,33,34]. Thus, it was shown [32] that hydrophobic fractions (mostly humic acids of soil origin) with molecular masses over 500 Da form halogenated products in significantly higher quantities than hydrophilic fractions (aquatic fulvic acids) with molecular masses below 500 Da. High levels of fulvic acids in dissolved organic matter (DOM) results in more brominated than chlorinated THM during aquatic chlorination [8]. Seasonal changes in the composition of DOM may lead to notable changes in the composition of DBPs [6,17], inspiring an active search for efficient methods of elimination of fulvic and humic acids from water as the main precursors of DBPs [35,36,37,38].

Bromide anions, being natural water components, interact with “active chlorine” and, depending on pH, form hypobromic acid (HOBr) and/or corresponding hypobromite anions (OBr^−^) [39,40]. Both species readily react with dissolved organic compounds. Moreover, HBrO/BrO^−^ is significantly more reactive towards DOM (*k_app_* = 1.6 × 10^6^ M^−1^ s^−1^) than HClO/ClO^−^ (*k_app_* = 41 M^−1^ s^−1^) [41]. Interestingly, THM levels increase in the presence of ppb levels of bromides and ppm levels of chlorides during aqueous chlorination of sugars [42]. The presence of bromides below 100 ppb results in their complete disappearance and formation of the corresponding brominated DBPs. Some earlier publications deal with studies of aqueous chlorination of water enriched with technogenic impurities, ions of iron and bromide [43,44].

It is worth mentioning that disinfecting agents themselves may be treated as a source of bromides. Thus, phenol was used as a trap in experiments on aquatic chlorination in deionized water without even traces of bromides [45]. Nevertheless, reactions with aqueous chlorine used to produce drinking water resulted in the formation of bromodichloro-, dibromochloro- and tribomophenol, together with the expected trichlorophenol, demonstrating the presence of brominating agents in the aqueous chlorine. The reason involves the fact that NaBr is a natural impurity in NaCl used in the production of industrial chlorinating reagents based on active chlorine, e.g., sodium hypochlorite. Since the reaction rate of hypobromic acid with aromatic species is significantly higher in comparison with hypochloric acid [41], even traces of bromine in technical chlorinating reagents may result in notable levels of the corresponding brominated DBPs [46,47].

The formation of organobromines and their higher impact than that of the corresponding organochlorines [7,14,48,49,50] require conducting targeted monitoring with quantification of the known ones and non-targeted screening of all types of these compounds to identify novel ones. Quite often, quantification of the most toxic individual organobromines is accompanied by the estimation of the overall content of brominated compounds, similarly to other integrated parameters. These parameters are quite important for the express testing of the safety of water. The following indices are the most demanded nowadays: total organic carbon (TOC), dissolved organic carbon (DOC), biodegradable dissolved organic carbon (BDOC), ultraviolet absorption (A_254_), specific ultraviolet absorption (SUVA = A_254_/TOC), dissolved organic nitrogen (DON) and total organic halogen (TOX) [51,52,53,54]. An alternative approach involves measuring the total content of organohalides (chlorides, bromides, iodides) by ion chromatography after complete sample destruction [55,56,57]. These integral indices are called total organic bromine (TOBr), total organic chlorine (TOCl) and total organic iodine (TOI) and represent constituents of TOX. It is worth emphasizing that that indices estimate the value of extractable halogenated DBPs from water with organic solvents, including volatile, semi-volatile and non-volatile compounds. All of them are rather hazardous. Volatile and semi-volatile species are highly biopermeable. They notably decrease the organoleptic properties of drinking water. Many of them are regulated all over the world. Higher-mass non-volatile DBPs may also lead to volatile and semi-volatile species while degrading. Moreover, they can also be harmful for humans, while their structures and properties are studied much less than those of the more volatile species. Therefore, to understand the pathways of the aqueous chlorination of natural water containing DOM, to calculate the balance of the addition of disinfecting agents, and to produce drinking water of better quality, it is necessary to monitor as many individual DBPs as possible, taking into account their precursors and mechanisms of their formation and transformation.

To differentiate the types of DBP in everyday activity of Ufavodokanal—a water treatment station in Ufa (Russia)—the following integrated indices of water quality were developed and introduced into practice: technogenic organic carbon (TgOC), technogenic organic chlorine (TgOCl), technogenic organic bromine (TgOBr) and technogenic organic oxygen (TgOO) in semi-volatile organic compounds (SVOCs) with boiling points in the range of 150–500 °C, isolated from water by liquid–liquid extraction [58].

The present study covers the results of long-term monitoring of numerous regulated volatile DBPs, including trihalometanes (THMs) and haloacetic acids (HAAs), as well as principal halogenated semi-volatile organic compounds in water intakes and drinking water of Ufa City (Russia). The study aims at the clarification of the formation of volatile and semi-volatile chlorinated and brominated DBPs during drinking water preparation in water intakes of various types of an industrial megapolis and the discovery of the most important factors influencing their formation. Non-volatile halogenated species are also taken into consideration in the research. Since the results of over 10 years of monitoring were analyzed, it was possible to process the data using various statistical approaches. 

## 2. Results and Discussion

The major halogenated SVOCs extracted from water at pH 2 from the samples are listed in Table 1.

These compounds were not detected in the samples of natural water from both water intakes. However, they were always present in the samples after chlorination. Extraction at pH 10 did not allow the detection of other SVOCs in measurable concentrations. The levels of all the detected DBPs were measured as weight and molar units. On the basis of these measurements, the overall content of elements in semi-volatile organic products was calculated (Table 2).

**Figure 1:**
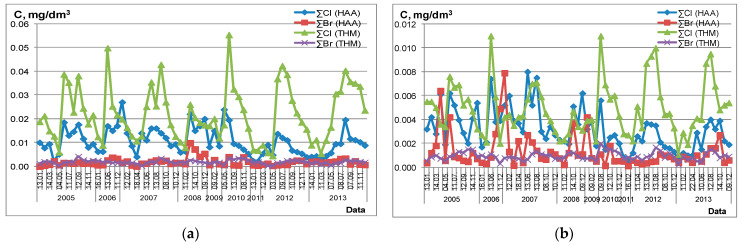
Integral content of chlorine and bromine in THM and HAA in the drinking water of the surface intake (**a**) and infiltration intake (**b**) in 2005–2013.

According to the obtained results, volatile DBPs (THM and HAA) were present in the analyzed samples at similar levels, while organochlorine species dominate (Table 2, Figure 1). Earlier, it was shown that chloroform, dichloro- and trichloro-acetic acids are the main products of aqueous chlorination [59,60,61,62]. According to the averaged results (2002–2019), bromine constituted about 7% of THM in surface water intake (SW) and 13% in infiltration water intake (IW), while chlorine was 83% and 79%, respectively. Bromine also constituted 9% of HAA in SW and 13% in IW, while chlorine was 55% and 47%, respectively. The results are quite logical, as THM mainly forms due to haloformic reactions of HAA [63]. 

**Figure 2:**
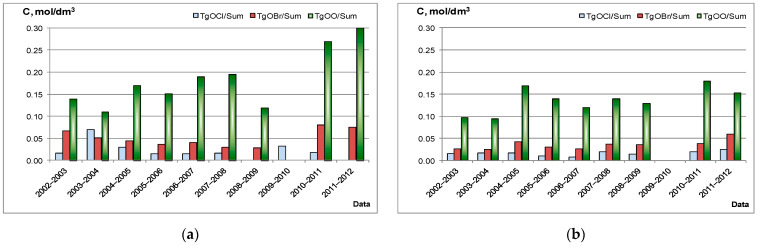
Annual averaged values of oxygenated, chlorinated and brominated SVOCs in total SVOCs in the drinking water of surface (**a**) and infiltration (**b**) intakes in 2002–2012. Some data are absent due to technical problems in that particular year.

**Figure 3:**
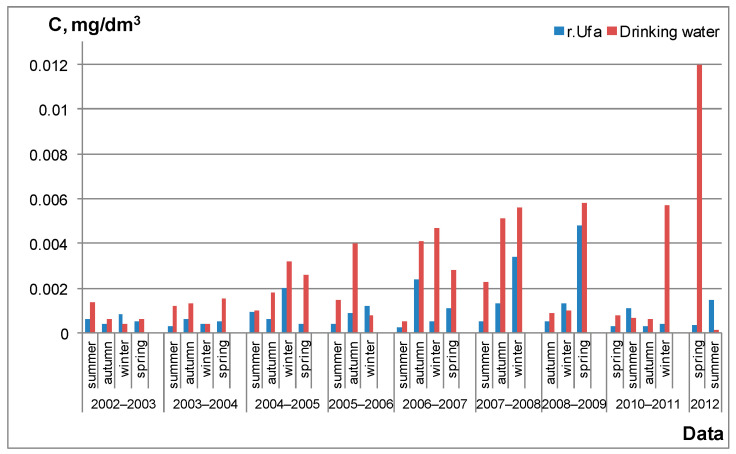
Seasonal values of TgOO, mg/dm^3^, in the water of the Ufa River and drinking water of SI.

Nevertheless, a reverse tendency was discovered for halogenated SVOCs. The levels of organobromine SVOCs are higher than that of organochlorine ones (Table 2). In the weight units, the levels of bromine are 5.5 times higher than that of chlorine in SW and 7.0 times higher in IW. In molar concentrations, reflecting the quantity of bromine and chlorine atoms in the formed SVOC molecules in the water volume unit, TgOBr dominates over TgOCl by a factor of 2.5 for SW and 3.2 for IW. These results are confirmed by the long-term monitoring (Figure 1). Taking into account the much higher reaction rate of hypobromic acid with aromatic moieties [41] and, at the same time, its lower oxidation potential, it is possible to propose that active bromine reacts very quickly with the aromatic fragments of DOM, while active chlorine, present in much higher quantities, starts oxidizing all biological and organic molecules. Bromine remains in higher-mass species which slowly decompose further with active chlorine, producing volatile and semi-volatile halogenated DBPs. Since numerous reaction steps involving active chlorine are required to produce VOCs, the content of chlorine vs. bromine in VOCs is higher than in SVOCs. It is also worth mentioning a notable contribution of TgOO to the composition of SVOCs (14–16%), demonstrating the decisive role of oxidative destruction in the formation of halogenated SVOCs. That conclusion can be supported by the long-term data summarized in Figure 2 and Figure 3. Since aqueous chlorination may involve electrophilic addition, electrophilic substitution, oxidation and even radical substitution [64], direct halogenation of hydrocarbons, especially of those containing double bonds and aromatic rings, is theoretically possible. However, corresponding hydrocarbons (possible precursors) were not present in the natural water before the chlorination. Only traces of xylenes were sometimes detected. Therefore, hydrocarbons cannot be the main precursors of the organohalides listed above and the presence of the latter in chlorinated water allows for considering them as oxidative destruction products. The higher levels of organobromines of all types (Table 2) in IW samples are observed due to the higher bromide content in the infiltration water (up to 32.6 ± 9.8 µg/dm^3^ in the river and up to 60.4 ± 18.1 µg/dm^3^ in the wells). 

**Figure 4:**
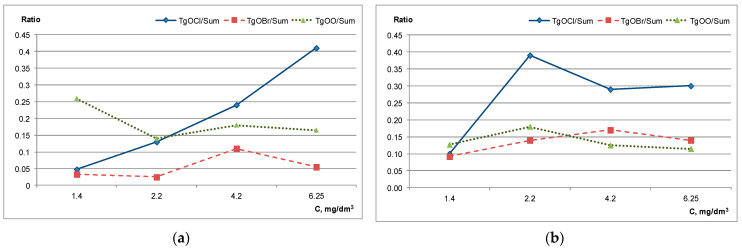
The ratios of chlorinated, brominated and oxygenated SVOCs in total SVOCs vs. reacted active chlorine concentration during laboratory experiments with surface water intake (SW) (**a**) and infiltration water intake (IW) (**b**).

To understand the dynamics of changes of brominated and chlorinated SVOCs, laboratory experiments with water samples from both intakes were conducted. River water was rather rich in DOC (4.8 mg/dm^3^), and for the infiltration water, that value was 0.8 mg/dm^3^. Reactions with chlorine water were carried out with various chlorine doses (1.4–6.25 mg/dm^3^, 4 h for river water and 0.5–0.85 mg/dm^3^, 2 h for infiltration water). These parameters were close to those used at the drinking water station. Figure 4 shows the higher levels of chlorinated SVOCs over brominated ones. However, there are differences between SW and IW. The maximum was not reached on the curve of SW chlorination. Chlorinated SVOC levels increase constantly with time, demonstrating that the concentration of DOM is rather high and that active chlorine reacts with DOM, forming primary, secondary, tertiary, etc. products. In the case of IW, the curve demonstrating chlorinated SVOC levels passed the maximum at an active chlorine concentration of 0.58 mg/dm^3^ (Figure 4b). At that stage, the highest level of SVOCs was probably reached, while additional active chlorine only transformed SVOCs into VOCs.

The levels of brominated SVOCs passed the maximum for both types of water. That maximum corresponds to rather high levels of applied active chlorine. Moreover, at the initial stage of the experiment, brominated SVOCs were not detected in the experiments with river water. Most probably, all bromine present in the water and the chlorinated agent rapidly reacted with DOM, resulting in the formation of heavy non-volatile molecules, without the rupture of C-C bonds. On the contrary, chlorine slowly reacted by mechanisms of addition, substitution or oxidation. That is why the longer reaction time results in higher TgOCl values. Moreover, chlorine destroys higher molecular mass compounds (including brominated ones) with formation of the corresponding SVOCs. It is possible to mention, once again, that there were more brominated species among SVOCs in infiltration water due to higher bromide levels in the wells. The lower levels of brominated SVOCs at the highest levels of active chlorine may involve further transformation of these compounds, formed earlier, into the volatile species.

Since bromides were not introduced in the laboratory experiments, chlorinated SVOCs dominated in the reaction mixture while, in real drinking water, brominated species dominated. This result may be also rationalized by the impossibility to reproduce the real conditions of industrial disinfection in the laboratory. For example, in laboratory experiments, there are no algae and other microflora growing in reservoirs of fresh water where chlorination takes place at the water treatment station. A notable portion of the chlorinating agent is wasted interacting with these species.

A technologically applied dose of the chlorinating agent directly depends on the quantity of microbiota and DOM. Due to higher levels of these parameters in the river water, the dose and, correspondingly, the levels of DBPs, are higher for SW. Nowadays, from the epidemiological point of view, the crucial parameter is the quantity of the remaining chlorine, rather than the dose of the chlorinating agent, as transportation of the drinking water along the distribution network should also be taken into account. However, at higher DOM levels, due to seasonal or other factors, that approach can result in increased levels of DBPs and especially halogenated SVOCs.

### 2.1. Chemometric Approaches

To find crucial factors influencing DBP formation, correlation analysis (interdependence check of each variation of two variables [65]) of the data collected over 17 years was carried out. The absence of high values of the statistically significant correlation coefficients (CCs), highlighted in Table 3, in some cases proved that not all the studied parameters demonstrated linear correlations with water quality characteristics.

Thus, VOC and SVOC types of DBPs showed quite different levels of correlation with the overall active chlorine dose: ƩTHM/chlorine dose (CC 0.64); ƩHAA/chlorine dose (CC 0.28); ƩHal-SVOC/chlorine dose (CC 0.15). The latter value signifies the absence of linear correlation. On the other hand, THM formation depends similarly on the primary and secondary chlorine doses (CC 0.67 and 0.51, respectively), while HAA formation mainly depends on the secondary one (CC 0.37 and 0.59). Therefore, THM formation presumably involves readily oxidizable DOM moieties. On the contrary, HAA formation requires higher chlorine doses and involves less reactive fragments of DOM. Generally, the correlation between Hal-SVOC formation and active chlorine doses is weak, allowing for concluding that some other parameters of water quality may be more important. THM and HAA formation have a reasonable correlation (CC 0.55), which is quite reasonable, as HAAs are the main precursors of THM. On the contrary, a linear correlation between the levels of VOCs and SVOCs is not observed. The absence of a linear correlation between SUVA_river_ and SUVA_drinking water_ proves that aqueous chlorination is mainly the process of DOM oxidative destruction completely changing the ratio A_254_/DOC.

**Scheme 1:**
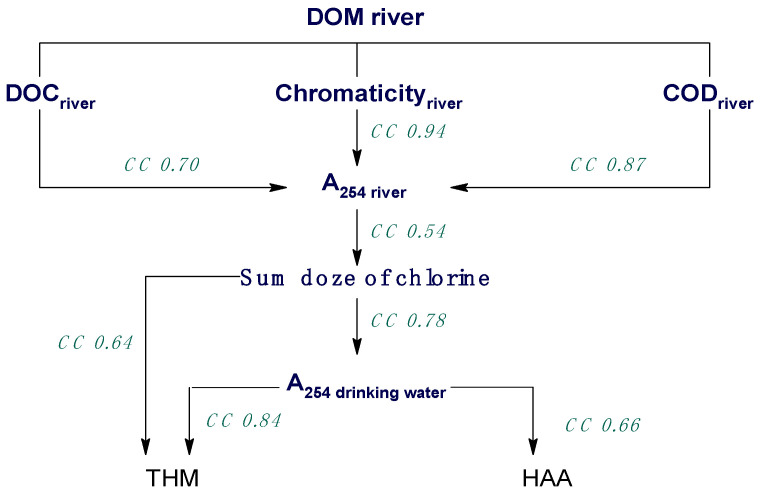
Correlation between river water quality indicators, the dose of the chlorinating agent and the volatile chlorination by-products: DOM—dissolved organic matter, DOC—dissolved organic carbon, COD—chemical oxygen demand, A_254 drinking water_—optical density at 254 nm, THM—trihalomethanes, HAA—haloacetic acids.

In general, there is no direct influence of the traditional integral parameters of the natural water quality on the formation of volatile DBPs. The crucial point is the dose of the chlorinating agent and A_254_. The latter represents the only parameter demonstrating at least a weak correlation both for river and drinking water (Table 3, Scheme 1). Moreover, any correlations are totally absent for SVOCs.

The absence of the direct correlations between THM, HAA and SUVA of the treated water was mentioned previously [53]. However, the authors emphasized that that is applicable only for natural water with low values of SUVA and UV absorption, as SUVA does not cover reactive sites in DOM responsible for the formation of DBPs. Nevertheless, studying chloroamination [66], the authors obtained linear correlations between SUVA and the formation of dichloroacetic acid (CC 0.82) and chloroform (CC 0.73), proving the decisive influence of the matrix and nature of the chlorinating agent.

Chlorine quantity in volatile DBPs (ƩCl in THM and HAA) notably correlates with the A_254_ value in drinking water (CC 0.67–0.85), and to a lesser extent with A_254_ value in the river water (CC 0.39–0.42). On the contrary, bromine quantity (ƩBr in THM and HAA) linearly correlates only with DOC_river_ and SUVA_river_ (CC 0.35–0.41). A negative correlation coefficient demonstrates that these variables are inversely proportional.

**Figure 5:**
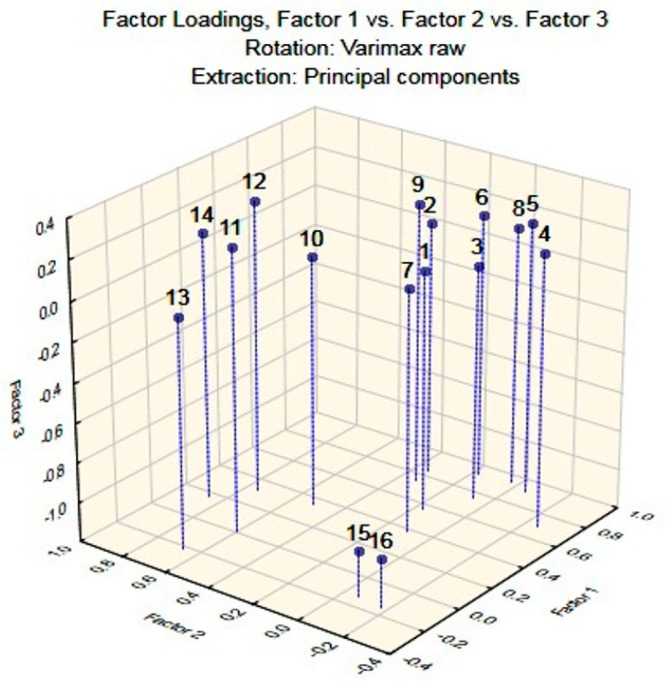
Results of the PCA analysis of the parameters related to the river and drinking water demonstrating three separate groups. 1. primary chlorine dose, 2. secondary chlorine dose, 3. COD (river), 4. turbidity (river), 5. color (river), 6. PMI (river), 7. DOM (river), 8. A_254_ (river), 9. DOC (drinking water), 10. A_254_ (drinking water), 11. Br level in HAA, 12. Cl level in HAA, 13. Br level in THM, 14. Cl level in THM, 15. TgOCl, 16. TgOBr.

Principal component analysis (PCA) is often used in multidimensional statistical analysis [59,67]. It allows combining two or even more variables into a single one (factor). That combination is based on the evaluation of the dispersion of variable values around a certain value, characteristic of the new factor, resulting in the unification of the closely related variables. An application of that approach allowed clustering of the used parameters into three groups: traditional indicators of water quality, semi-volatile DBPs and volatile DBPs (Figure 5).

Therefore, the results show that the variability of VOC and SVOC levels in aqueous chlorination does not depend on a single joint factor demonstrating linear correlation with these DBP classes. The conducted analysis allowed for separating the used parameters into three groups. The first one consists of the primary and secondary chlorine doses as well as general indices of water quality. VOC levels and A_254_ index constitute the second group, while the third one is represented exclusively by SVOC levels. Selection of a higher or lower number of factors does not allow merging of VOCs and SVOCs into a joint group. The value of factor load was never below 0.5.

### 2.2. Seasonal Peculiarities

**Figure 6:**
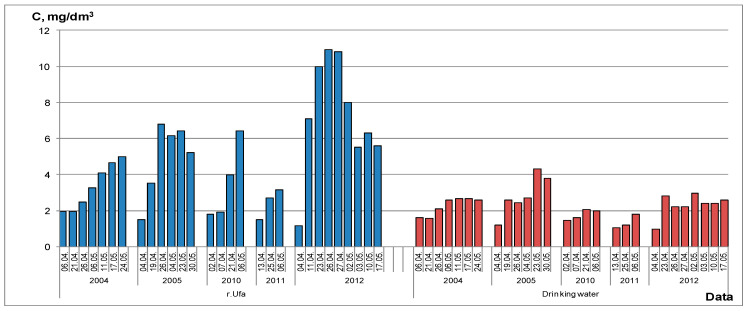
DOC levels in the Ufa River water and drinking SW during spring floods in 2004, 2005, 2010, 2011, 2012.

**Figure 7:**
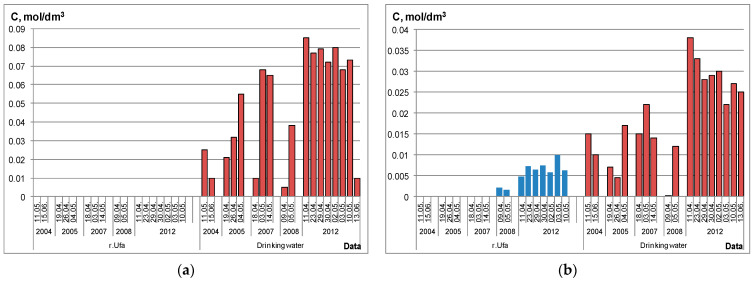
The ratio of TgOBr (**a**) and TgOCl (**b**) to the total SVOCs in the flood periods of 2003, 2004, 2005, 2007, 2008, 2012.

All the discussed results of the correlation analysis were based on the long-term monitoring with the exception of flood periods. Correlations for chromaticity/PMI, chromaticity/COD, turbidity/PMI, turbidity/COD and COD/PMI for the Ufa River increase, while the CC for the pairs DOM/turbidity, DOM/chromaticity and DOM/PMI do not change or even decrease during floods. These results may testify that organic compounds of different natures (i.e., soil flushes with melted snow) appear in river water [68]. A major portion of organic compounds composing DOM participate in continuous biotransformation processes in water and sediments, thus, DOM represents the most reactive component of aquatic ecosystems sensitive towards any changes of the external environment [69]. Figure 6 contains the data on DOC in the Ufa River and illustrates a possible sharp change in the water quality in the flood periods of 2004–2012. Thus, in 2012, DOC significantly increased in the Ufa River water, while drinking water possessed a persistent chemical smell, which could not be eliminated by changing the reagent doses during flocculation/coagulation procedures. Nevertheless, integral parameters, including COD and PMI, did not notably increase, and the levels of THM and HAA remained at their usual values. On the contrary, the levels of halogenated SVOCs increased (Figure 7). The latter fact allows for making the conclusion that additional DOM during floods consists of heavier organic structures not so easily oxidizable with the formation of VOCs.

Another important issue involves the winter period. Stirring up of sediments, especially during freezing, plays a significant role in the formation of chlorinated SVOCs. Sharp discharges of notable water volumes from the electric power station situated upstream relative to Ufa City change the hydraulic regime of the river below the ice, resulting in stirring up of the river sediments [70]. That event results in higher levels of SVOCs and a more pronounced smell. Nevertheless, the levels of THM and HAA remain at the usual level. Higher-mass natural products from sediments may not readily form halogenated VOCs, while the levels of SVOCs and non-volatile halogenated species increase. An additional smell may be rationalized by the formation of aliphatic species with lower reactivity towards active chlorine, e.g., alcohols, aldehydes.

**Figure 8:**
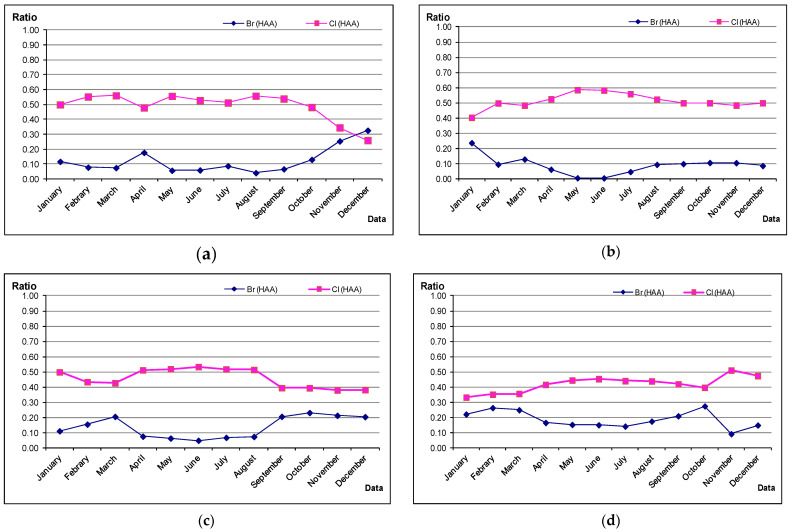
Relative content of Br and Cl in HAA in IW in 2006 (**a**), 2011 (**b**), 2012 (**c**), 2013 (**d**).

Another issue worth mentioning involves an increase in the bromine content in HAA in IW in the fall–winter period. Figure 8 illustrates corresponding results in the years when that effect was particularly pronounced. That issue may be rationalized by a higher percentage of underground water in the wells. It is highly mineralized in comparison with river water also penetrating the wells (up to 80% in summertime). The effect nicely correlates with the increase in the water hardness in these periods. River water does not demonstrate the mentioned seasonal effect.

The following calculations were conducted in order to reveal the material balance for Cl and Br, present in the natural water and added with chlorinating agents vs. detected in DBPs in the averaged long-term data. The left part of the equation representing chlorine contains chlorine from the chlorinating agent and natural inorganic chlorides in the river water. The right part contains chlorine of VOCs, SVOCs, and non-volatile DBPs as well as chlorine wasted for the disinfection and inorganic chlorides in drinking water. It was impossible to separate chlorine content in non-volatile compounds and wasted in disinfection (reactions with algae, microflora, inorganic species). Thus, the value was calculated as the difference between the right and left parts of the equation. Therefore, ƩC_Clnon-volatiles + disinfection_, equals 1.56 mg/dm^3^, i.e., about 95% of the introduced active chlorine.

C_Clchlorine agent_ + C_Clriver_ = (ƩC_ClTHM_ + ƩC_ClHAA_ + ƩC_ClSVOC_) + ƩC_Clnon-volatiles+disinfection_ + C_Cldrinking water_

1.59 + 7.8 = (0.0218 + 0.0095 + 0.00071) + ƩC_Clnon-volatiles+disinfection_ + 7.8

$$9.39\;=\;0.032\;+\;\Sigma C_{\mathrm{Clnon}\text{-}\mathrm{volatiles}+\mathrm{disinfection}}\;+\;7.8\underset{}{\Rightarrow }\Sigma C_{\mathrm{Clnon}\text{-}\mathrm{volatiles}+\mathrm{disinfection}}\;=\;9.39\;-\;0.032\;-\;7.8\;=\;1.56$$

Material balance for bromine may be represented by the following equation, while the general quantity of bromine in the river and drinking water, as well as in the disinfecting agent, was measured with inductively coupled plasma–mass spectrometry (ICP-MS). Since bromides were not present in the drinking water, all inorganic bromine finally appeared in the brominated DOM species. It is worth mentioning that about 75% of bromine is introduced as an impurity to the chlorinating agent.

C_Br in chlorinating agent_ + C_Br in river_ = (ƩC_Br in THM_ + ƩC_Br in HAA_ + ƩC_Br in SVOC_) + ƩC_Br non-volatile_

0.105 + 0.0326 = (0.00174 + 0.0017 + 0.0037) + ƩC_Br non-volatile_

$$0.138\;=\;0.00714\;+\;\Sigma C_{\mathrm{Br}\;\mathrm{non}\text{-}\mathrm{volatile}}\underset{}{\Rightarrow }\Sigma C_{\mathrm{Br}\;\mathrm{non}\text{-}\mathrm{volatile}\;}=\;0.131$$

The obtained value of ƩC_Br non-volatile_ = 0.131 mg/dm^3^ represents about 95% of the overall bromine introduced at the disinfection stage, meaning that in the case of complete chlorination, resulting in the full destruction of humic matter, it should appear in brominated VOC and SVOC species.

## 3. Materials and Methods

### 3.1. Sampling

The following samples were collected regularly in 2002–2019:

- drinking water of the surface water intake (SW). Water comes from the Ufa River and is subjected to multistage purification including UV irradiation, primary chlorination, coagulation with aluminum sulfate, flocculation with polyacrylamide, upholding, fast filtration through filters with baked clay and secondary chlorination;
- drinking water of the infiltration water intake (IW). Water comes from the infiltration wells and is subjected only to the single-stage disinfection with molecular chlorine;
- water of the River Ufa before and after aqueous chlorination in the laboratory with various doses of molecular chlorine;
- water from the joint collector at the infiltration water intake (well depth ~25 m, distance ~120 m from the river).

### 3.2. Sample Preparation

A water sample of 1 dm^3^ was saturated with NaCl and subjected to extraction with dichloromethane (1 × 60 mL) at pH 2. The extract was concentrated in a Kuderna-Danish to 1:20,000. All reagents were of high purity (Merck, Darmstadt city, Germany). Concentrated extracts were analyzed in parallel with GC-MS to identify SVOCs and gas chromatography with an atomic emission detector (GC-AED) to estimate general content of carbon-, chlorine-, and bromine-containing compounds using the proposed integrated indices of water quality.

GC-MS analysis was carried out with an Agilent 6890N-5973 instrument (Agilent, Santa Clara, CA, USA). Helium was used as a carrier gas with flow rate of 2 sm^3^/min in split (1:5) mode, injector temperature 250 °C, capillary column HP-5MS 30 m × 0.32 mm × 0.25 µm programmed from 30 °C (the initial temperature) without isotherm to 60 °C at 20 grad × min^−1^, then to 250 °C (final temperature) at 6 grad × min^−1^. The holding time at the final temperature was 10 min. Total analysis time was 47 min.

Mass spectra were recorded in electron ionization mode. Ion source temperature—230 °C; electron energy—70 eV; full mass spectra were recorded in the mass range 45–600 amu, Willey 138 and NBS spectral libraries were used for the identification. The hits were accepted if the score was above 700 and further confirmed manually using the known rules of fragmentation of organic compounds [71]. Quantification was carried out using dimethylphthalate-3,4,5,6-d_4_ and dioctylphthalate-3,4,5,6-d_4_ as internal standards. The procedure resembled to some extent the US EPA 8270 method.

The applied conditions allow detecting SVOCs with Kovats indices up to 3200 on non-polar phases, covering various technogenic ecotoxicants, low and middle molecular weight products of DOM destruction. Extension of the analysis time did not allow for detecting additional organic compounds.

The GC-AED system consisted of GC HP 6890 and AED G2350A HP instruments (Agilent, Santa Clara, CA, USA). Chromatographic columns and conditions were the same as described above for GC-MS analysis. The temperature of the resonator chamber was 250 °C, interface was 250 °C; helium purging flow rate (purge vent) in the resonator was 35 sm^3^/min; plasma-forming helium flow rate (cavity vent) in the resonator was 60.5 sm^3^/min; nitrogen flow rate to purge spectrometer was 400 sm^3^/min; entrance oxygen pressure was 196–245 kPa, hydrogen was 63–77 kPa, methane/nitrogen mixture was 210 kPa. Quantification with GC-AED was conducted using component-independent calibration with acylbromide, bromotetradecane, 2,4,6-tribromophenol, 2-btomo-3-methylbutene-2, 3-bromo-2,2-dimethylpropanol-1, 1-bromonaphthalene, 2,4-dichlorophenol, 2,4,6-trichlorophenol, α-HCCH, γ-HCCH, DDE as standards. Processing was based on the summarized signal of all peaks on the element selective chromatograms recorded at the following wave lengths: 193 nm for carbon, 171 nm for oxygen, 479 nm for chlorine and 478 nm for bromine. A summarized signal facilitated processing of the results for the rich matrices of natural samples [72].

Monitoring or technological studies of water quality are generally based on comparative analysis, i.e., decrease or increase in the levels of the targeted components after some changes in the process. Considering that SVOC composition remains more or less constant for the selected water intake and water preparation technology, when carrying out comparative studies, we did not take into account isolation factors of the individual compounds used for the calculation of TgOC, TgOCl, TgOBr and TgOO. Detection limits for these indices were as follows TgOC—1 × 10^−5^ mg/dm^3^, TgOCl and TgOBr—1 × 10^−6^ mg/dm^3^, TgOO—1 × 10^−4^ mg/dm^3^.

Quantification of HAA was conducted using gas chromatography with an electron capture detector after their methylation. The method is analogous to US EPA 552.2 and was adapted to the natural background, characteristic of the water intakes in the study. The method is validated and included in the list of the official methods of the Russian Federation (No 1.31.2011.09374).

A 1N NaOH solution was added to a water sample of 50 sm^3^ to achieve pH 11.5. Then, methyl-*tert*-butyl ester was used to extract all the interfering impurities from the sample. To extract the acids, the water sample was acidified to pH < 0.5 with sulfuric acid. The addition of 3 g of CuSO_4_·5H_2_O and 12 g of Na_2_SO_4_ was followed by energetic stirring to dissolve the salts. The targeted acids were extracted with 5 sm^3^ of methyl-*tert*-butyl ester. The obtained extract was introduced into a reaction with methanol solution with 10% sulfuric acid at 50 °C for 2 h. Then, the reaction mixture was cooled and neutralized with sodium bicarbonate. 1,2,3-Trichloropropane as an internal standard was added to a precise volume of the organic extract and the sample was injected into GC. A capillary column of medium polarity, OV-17 (30 m × 0.32 mm × 0.25 µm), was used for the analysis. The column temperature was programmed from 50 °C with a 14 min hold at this initial temperature to 145 °C at 3 grad × min^−1^, then to 210 °C (final temperature) at 20 grad·min^−1^.

THMs were quantified using the MEGA chromatograph and headspace approach with automatic sample preparation and HS 850 injection device (Fisons Instruments, Manchester, UK) followed by electron capture detection. An HP-624 capillary column (30 m × 0.25 mm × 1.4 µm) was used. The following parameters were used: sample volume—10 sm^3^; time in thermostat—15 min at 70 °C; syringe temperature—80 °C; injected vapor volume—1 sm^3^; transport channel temperature—140 °C; injector temperature—250 °C; detector temperature—300 °C; column temperature—40 °C (5 min) to 180 °C at 8 grad×min^−1^, then to 250 °C (0.17 min) at 30 grad×min^−1^; He flow rate 2 sm^3^/min. The method is officially certified in the Russian Federation (FR. 1.31.2008.04835).

Dissolved organic carbon (DOC) was measured according to the GOST 31958–2012 method using photometry after preliminary destruction of the organic carbon with UV irradiation in the presence of K_2_S_2_O_8_ as an oxidant to form carbon dioxide. The latter, penetrating through the gas membrane, was absorbed by the buffer solution of phenolphthaleine. The optical density of the solution was measured at a wavelength of 550 nm. A standard solution of potassium hydrophthalate was used for the calibration. A water sample was made to pass through a membrane filter of 0.45 µm. The method is analogous to ASTM 53 and officially certified in the Russian Federation (FR. 1.31.2013.16255).

The permanganate index (PMI) was measured according to PNDF 14.1:2:4.154–99 (analog of International Method ISO 8467) after the water sample treatment with potassium permanganate during boiling in acidic media. Detection limit of the method is 0.25 mg O/dm^3^.

COD was measured according to GOST 31859–2012 (analog of ISO 6060), based on the oxidation of organic compounds with potassium bichromate at elevated temperatures in acidic media. The detection limit of the method is 4 mg O/dm^3^.

Optical density at 254 nm (A_254_) was measured with a DR 5000 spectrophotometer (HACH, Berlin, Germany).

The specific ultraviolet absorption (SUVA) index was calculated as A_254_/TOC.

Measurements of total bromine were carried out using an Aurora Elite mass spectrometer (Bruker, Bremen, Germany) with inductively coupled plasma (ICP-MS). The calibration curve obtained with a standard solution of KBr covered the range 0.001–1 mg/dm^3^. Isotope ^79^Br was used for quantification carried out without any dilution for natural and drinking water samples and with 10-fold dilution in the case of active chlorine technical solutions. The instrument had the following parameters: RF power—1.60 kW, sampling depth—5.0 mm, plasma flow—18.0 L/min, auxiliary flow—1.65 L/min, sheath gas—0.23 L/min, nebulizer flow—0.80 L/min, dwell time—50 ms.

The statistical calculations were performed using the Statistica 10 software package [73].

## 4. Conclusions

Estimation of chlorinated and brominated DBP formation based on long-term monitoring (2002–2019) of drinking water quality from surface and infiltration intakes in Ufa (Russia) showed that the levels of volatile THM and HAA are quite similar throughout the year (±10–15%), with the prevalence of chlorinated species. Bromine level in THM in SW constitutes ~7% and in IW, 13%. For chlorine, these values are 83% and 79%, respectively. HAAs in SW contain 9% and in IW 13% bromine and 55% and 47% chlorine, respectively. However, SVOCs demonstrate a reverse trend. Brominated species (bromopropanone-2,2-methyl-3-bromobutanol-2,1-bromo-2-methylbutanol-2, etc.) dominate over the chlorinated ones in the SW by a factor of 5.5 and in IW, a factor of 7. A notable contribution of oxygen in the total composition of SVOCs (14–16%) in both SW and IW confirms an important role of the oxidative destructive processes involving DOM during water chlorination. That process results in formation of the halogenated species from the non-volatile precursors. The levels of the dominant brominated SVOCs are higher than that of the chlorinated ones due to rather high levels of natural bromides in the natural water (~25%) and hypobromic acid in technical disinfection reagents (~75%). That bromine rapidly reacts with DOM, forming high molecular weight brominated DOM species without breaking C-C bonds. Actually, all available bromine penetrates organic substrates in the water disinfection conditions. Although reactions of organic matter with hypochloric acid are significantly slower, they involve the oxidation and formation of smaller organic molecules of non-volatile and semi-volatile DBPs, where bromine content is still quite high. Formation of VOCs from the original DOM, brominated DOM or SVOCs requires several additional reaction steps, including C-C bond cleavages. In the most cases, only active chlorine is available for these processes, resulting in the higher levels of chlorine rather than bromine in THM and HAA. It is also worth mentioning that about 95% of bromine in the drinking water belongs to non-volatile DOM species.

The application of correlation analysis and PCA to the data for 2002–2019 demonstrated notable linear correlations of THM and HAA with chlorine dose and further indirect correlations with the main indices of water quality: DOM, COD, PMI in river water →optical density A_254_ of the river →chlorine dose → THM, HAA. Nevertheless, correlations of chlorinated and brominated THM and HAA are different. On the contrary, there is no correlation of the halogenated SVOCs with water quality parameters or chlorine dose. PCA allowed for placing these compounds into a separate group. This fact does not eliminate the possibility of the presence of some non-linear correlations. Alternatively, some crucial parameter of the water quality might not be taken into account. It could involve structural peculiarities of DOM molecules characteristic of each particular water intake. It was shown that the disturbance of sediments, changes of the hydraulic regime of the river and soil flushes during floods can result in higher formation of the brominated SVOCs due to the changes of DOM composition. However, even in these cases, the levels of HAA and THM may demonstrate their usual levels. Stability of the basic parameters of drinking water preparation and intrariver parameters over several years rationalize the stability of the DBP composition and contributions of elements to their composition.

## Figures and Tables

**Table 1:** Semi-volatile organic compounds regularly present in the drinking water of Ufa City.

Name	CAS	Characteristic Ions in the Mass Spectra (*m*/*z* (Intensity))
1-Bromopropanone-2	598-31-2	43 (100); 136 (12); 138 (11)
2-Methyl-4-bromobutene-1	20038-12-4	69 (100); 41 (60); 55 (25))
1,1-Dimethyl-3-chloropropanol-1	1985-88-2	59 (100); 43 (71); 107 (25)
2,3-Dichloro-2-methylbutane	507-45-9	77 (100); 41 (51); 76 (33)
1,1,1-Trichloropropanone-2	918-00-3	43 (100); 125 (11); 127 (7)
2-Methyl-3-bromobutanol-2	2588-77-4	59 (100); 151 (15); 153 (14)
1,1-Dibromopropanone-2	867-54-9	43 (100); 173 (64); 216 (44)
1-Bromo-2-methylbutanol-2	31736-62-6	73 (100); 57 (36); 137 (29)
1,2,3-Trichloropropene-1	13116-57-9	109 (100); 144 (20); 146 (15)
1,3-Dibromopentane	42474-20-4	69 (100); 149 (77); 151 (74)
1-Bromo-2,3-dichloropropane	33037-07-9	111 (100); 75 (97); 113 (65)
1,4-Dibromopentanol-3	159475-15-7	137 (100); 139 (92); 57 (63)
1-Bromo-2,4-dimethylbenzene	583-70-0	105 (100); 184 (61); 186 (60)
3,5-Dibromopentanol-2	1000288-19-4	43 (100); 120 (56); 165 (28)

**Table 2:** Technogenic organic carbon (TgOC), Technogenic organic chlorine (TgOCl), Technogenic organic oxygen (TgOO) and Technogenic organic bromine (TgOBr) values for trihalomethanes (THM), haloacetic acids (HAA) and semi-volatile organic compounds (SVOCs) in drinking water from surface and infiltration water intakes averaged for 2002–2019.

Classes of Disinfection by-Products DBP	Value in Drinking Water							
	Surface Intake				Infiltration Intake			
	mg/dm^3^	%	mole/dm^3^	%	mg/dm^3^	%	mole/dm^3^	%
**Trihalomathanes**								
ƩC in THM	0.0025	9.6	0.00021	19.1	0.00049	7.4	0.000041	16.4
ƩCl in THM	0.02189	82.9	0.00062	56.4	0.0052	78.8	0.00015	60
ƩBr in THM	0.00175	6.6	0.000022	2.0	0.00087	13.2	0.000011	4.4
ƩTHM	0.02614		0.0011		0.0066		0.00025	
**Haloacetic Acids**								
ƩC in HAA	0.0025	14.5	0.00024	24	0.00076	16.9	0.000063	25.0
ƩO in HAA	0.0033	19	0.00024	24	0.0010	22.2	0.000063	25.0
ƩCl in HAA	0.011	55	0.00031	31	0.0021	46.7	0.000059	23.3
ƩBr in HAA	0.0018	9	0.000023	2.3	0.0006	13.3	0.0000075	3.0
ƩHAA	0.019		0.0010		0.0045		0.00025	
**Halogenated SVOC**								
TgOC	0.0083	55.3	0.00069	75.8	0.0063	65.0	0.00052	81.3
TgOO	0.0025	16.7	0.00015	16.5	0.0015	15.5	0.000093	14.5
TgOCl	0.00069	4.6	0.000019	2.5	0.00024	2.4	0.0000067	1.0
TgOBr	0.0038	25.3	0.000047	5.2	0.0017	17.1	0.000022	3.2
ƩSVOC	0.015		0.00091		0.0097		0.00064	

**Table 3:** Data of correlation analysis for integral parameters of drinking water quality of the surface water intake and the Ufa River for the period 2002–2019 (without floods), *p*-values < 0.05. The most significant statistical correlation coefficients are highlighted.

		Drinking Water													
		Sum SVOC	TgOC	TgOBr	TgOCl	TgOO	Sum HAA	Br in HAA	Cl in HAA	Sum THM	Br in THM	Cl in THM	DOC	A_254_	SUVA
**Drinking water**	Sum SVOC	1.00													
	TgOC	0.99	1.00												
	TgOBr	0.92	0.83	1.00											
	TgOCl	0.72	0.69	0.80	1.00										
	TgOO	0.95	0.91	0.92	0.66	1.00									
	Sum HAA	−0.16	−0.14	−0.20	−0.21	−0.16	1.00								
	Br in HAA	−0.18	−0.17	−0.18	−0.13	−0.18	0.60	1.00							
	Cl in HAA	−0.12	−0.11	−0.18	−0.21	−0.16	0.98	0.45	1.00						
	Sum THM	−0.25	−0.24	−0.29	−0.03	−0.31	0.55	0.22	0.55	1.00					
	Br in THM	−0.20	−0.22	−0.28	−0.08	−0.28	0.22	0.26	0.17	0.56	1.00				
	Cl in THM	−0.24	−0.24	−0.29	−0.03	−0.31	0.56	0.21	0.55	0.99	0.52	1.00			
	DOC	0.03	0.03	0.2	0.08	0.02	0.16	−0.13	0.16	0.40	−0.15	0.43	1.00		
	A_254_	−0.23	−0.23	−0.24	−0.23	−0.22	0.66	−0.19	0.67	0.84	0.18	0.85	0.15	**1.00**	
	SUVA	−0.28	−0.27	−0.34	−0.40	−0.27	0.07	0.18	0.02	0.28	−0.05	0.29	−0.50	**0.76**	**1.00**
**Tech**	I dose of Cl_2_	−0.13	−0.12	−0.14	0.1	−0.20	0.37	−0.04	0.42	0.67	0.19	0.68	0.48	0.44	−0.05
	II dose of Cl_2_	−0.21	−0.22	−0.19	−0.07	−0.18	0.59	0.15	0.62	0.51	0.06	0.52	0.64	0.67	0.01
	Sum dose of Cl_2_	−0.15	−0.14	−0.14	0.03	−0.15	0.28	−0.01	0.30	0.64	0.18	0.65	0.65	0.78	0.12
**River water**	Turbidity	0.01	0.01	0.01	0.01	−0.02	−0.17	−0.15	−0.14	−0.28	−0.45	−0.28	0.27	−0.24	−0.44
	Chromaticity	0.06	0.06	0.11	0.16	0.07	0.27	−0.05	0.31	0.07	−0.42	0.11	0.54	0.31	−0.21
	Permang. index	0.10	0.09	0.08	0.08	0.12	0.21	0.04	0.23	0.08	0.09	0.09	0.34	0.14	−0.22
	COD	0.22	0.21	0.21	0.12	0.23	0.08	−0.14	0.12	−0.04	−0.41	−0.03	0.11	0.50	−0.15
	DOC	0.17	0.16	0.22	0.21	0.16	0.28	0.41	0.27	−0.17	−0.40	−0.15	0.21	0.13	−0.10
	A_254_	−0.27	−0.31	−0.19	−0.08	−0.25	0.34	−0.09	0.39	0.39	−0.43	0.42	0.25	0.51	−0.03
	SUVA	−0.78	−0.80	−0.72	−0.65	−0.78	0.14	0.35	0.04	0.17	−0.37	0.19	−0.07	0.18	0.21
		**Drinking Water**													
		**Sum Dose of Cl_2_**	**Turbidity**	**Chromaticity**	**Permang. Index**	**COD**	**DOC**	**A_254_**	**SUVA**						
**Tech**	I dose of Cl_2_														
	II dose of Cl_2_														
	Sum dose of Cl_2_	1.00													
**River water**	Turbidity	0.09	1.00												
	Chromaticity	0.38	0.56	1.00											
	Permang. index	0.25	0.24	0.27	1.00										
	COD	0.09	0.22	0.39	0.20	1.00									
	DOC	−0.12	0.24	0.45	0.06	0.21	1.00								
	A_254_	0.54	0.39	0.94	0.23	0.87	0.70	1.00							
	SUVA	0.38	0.13	0.52	0.07	0.42	−0.08	0.62	1.00						

SUVA—specific ultraviolet absorption index was calculated as A_254_/Total organic carbon.

## Data Availability

The data presented in this study are available by the request to the authors.

## Sample Availability

Samples of the compounds are not available from the authors. Any more, being totally wasted.
